# Genome Sequence of the Edible Cultivated Mushroom *Lentinula edodes* (Shiitake) Reveals Insights into Lignocellulose Degradation

**DOI:** 10.1371/journal.pone.0160336

**Published:** 2016-08-08

**Authors:** Lianfu Chen, Yuhua Gong, Yingli Cai, Wei Liu, Yan Zhou, Yang Xiao, Zhangyi Xu, Yin Liu, Xiaoyu Lei, Gangzheng Wang, Mengpei Guo, Xiaolong Ma, Yinbing Bian

**Affiliations:** 1 Institute of Applied Mycology, Plant Science and Technology College, Huazhong Agricultural University, Wuhan, Hubei, China; 2 Key Laboratory of Agro-Microbial Resource Comprehensive Utilization, Ministry of Agriculture, Huazhong Agricultural University, Wuhan, Hubei, China; 3 Food Science and Technology College, Huazhong Agricultural University, Wuhan, Hubei, China; USDA Forest Service, UNITED STATES

## Abstract

*Lentinula edodes*, one of the most popular, edible mushroom species with a high content of proteins and polysaccharides as well as unique aroma, is widely cultivated in many Asian countries, especially in China, Japan and Korea. As a white rot fungus with lignocellulose degradation ability, *L*. *edodes* has the potential for application in the utilization of agriculture straw resources. Here, we report its 41.8-Mb genome, encoding 14,889 predicted genes. Through a phylogenetic analysis with model species of fungi, the evolutionary divergence time of *L*. *edodes* and *Gymnopus luxurians* was estimated to be 39 MYA. The carbohydrate-active enzyme genes in *L*. *edodes* were compared with those of the other 25 fungal species, and 101 lignocellulolytic enzymes were identified in *L*. *edodes*, similar to other white rot fungi. Transcriptome analysis showed that the expression of genes encoding two cellulases and 16 transcription factor was up-regulated when mycelia were cultivated for 120 minutes in cellulose medium versus glucose medium. Our results will foster a better understanding of the molecular mechanism of lignocellulose degradation and provide the basis for partial replacement of wood sawdust with agricultural wastes in *L*. *edodes* cultivation.

## Background

*Lentinula edodes*, also known as Xianggu or shiitake, belonging to the Agaricales order of the Agaricomycetes class in Basidiomycota phylum, is one of the white-rot fungi that grow on the dead tree or sawdust by degrading cellulose, hemicellulose and lignin. Meanwhile, *L*. *edodes* is the second most widely cultivated mushroom species all over the world, only second to *Agaricus bisporus* [1–3]. As a delicious edible mushroom initially cultivated more than eight centuries ago [4], *L*. *edodes* possesses plenty of proteins and polysaccharides as well as unique flavor components, which render shiitake overwhelmingly popular with consumers in Asian countries such as China, Japan and Korea. It is noteworthy that the immunomodulatory activities of polysaccharides and the function on calcium supplementation from *L*. *edodes* have been verified [4–7].

*L*. *edodes* can secrete plenty of lignocellulolytic enzymes capable of efficiently degrading lignin and cellulose, demonstrating that such enzymes could be applied in biotransformation and fiber bleaching as well as bioremediation [5,8,9]. In Asia, the main cultivation materials of *L*. *edodes* are hard wood or its sawdust. In the cultivation of *L*. *edodes*, the sawdust normally accounts for 80% of the total amount of the cultivation media [10]. However, some wood-rot fungi, such as *Pleurotus ostreatus* and *Flammulina velutipes*, can be cultivated with wheat straw, rice straw, corncob, cottonseed hull or bagasse as the main cultivation substrate [11,12]. This artificial sawdust cultivation mode of *L*. *edodes* may damage forest conservation. Currently, *L*. *edodes* cultivation is growing in occurrence in Australia, America, Europe and the Latin American countries [10,13,14], indicating the necessity to protect forest resources, reduce the production cost and shorten the culture cycle of *L*. *edodes* by replacing wood or sawdust with agricultural straws for cultivation of the species. To this end, we need to understand the genetic features of *L*. *edodes* in degrading lignocelluloses.

The composition of genes and the mechanisms of lignocellulose degradation are complex in wood-rot fungi [15]. Besides, the expression of these genes degrading cellulose, hemicellulose, pectin and lignin can be induced by various cultivation media. Additionally, the extracellular enzyme activity and substance degrading ability would be affected by various factors during the growth of the fungus [16]. Genome sequences of some edible fungi, including *A*.*bisporus*, *Ganoderma lucidum*, *F*. *velutipes*, *P*. *ostreatus* and *Volvariella volvacea*, have been completed recently [17–20], indicating that there is some relevance between the component of the lignocellulolytic enzyme gene and the variety of the cultivation materials in these edible mushrooms. To date, some studies associated with *L*. *edodes* lignin degrading enzymes have been reported [21,22], and an integrated genetic linkage map of *L*. *edodes* was constructed [23]. In this article, we report a draft genome sequence of monokaryotic *L*. *edodes* strain W1-26, and the identification of a large set of genes and potential gene clusters involved in lignocellulose degradation. Understanding the *L*. *edodes*’ capacity to degrade lignocellulose may facilitate the development of more effective strategies to degrade lignocellulosic feed stocks and help to improve the efficiency of edible mushroom cultivation.

## Results

### Genome sequencing and general features

**Fig 1:**
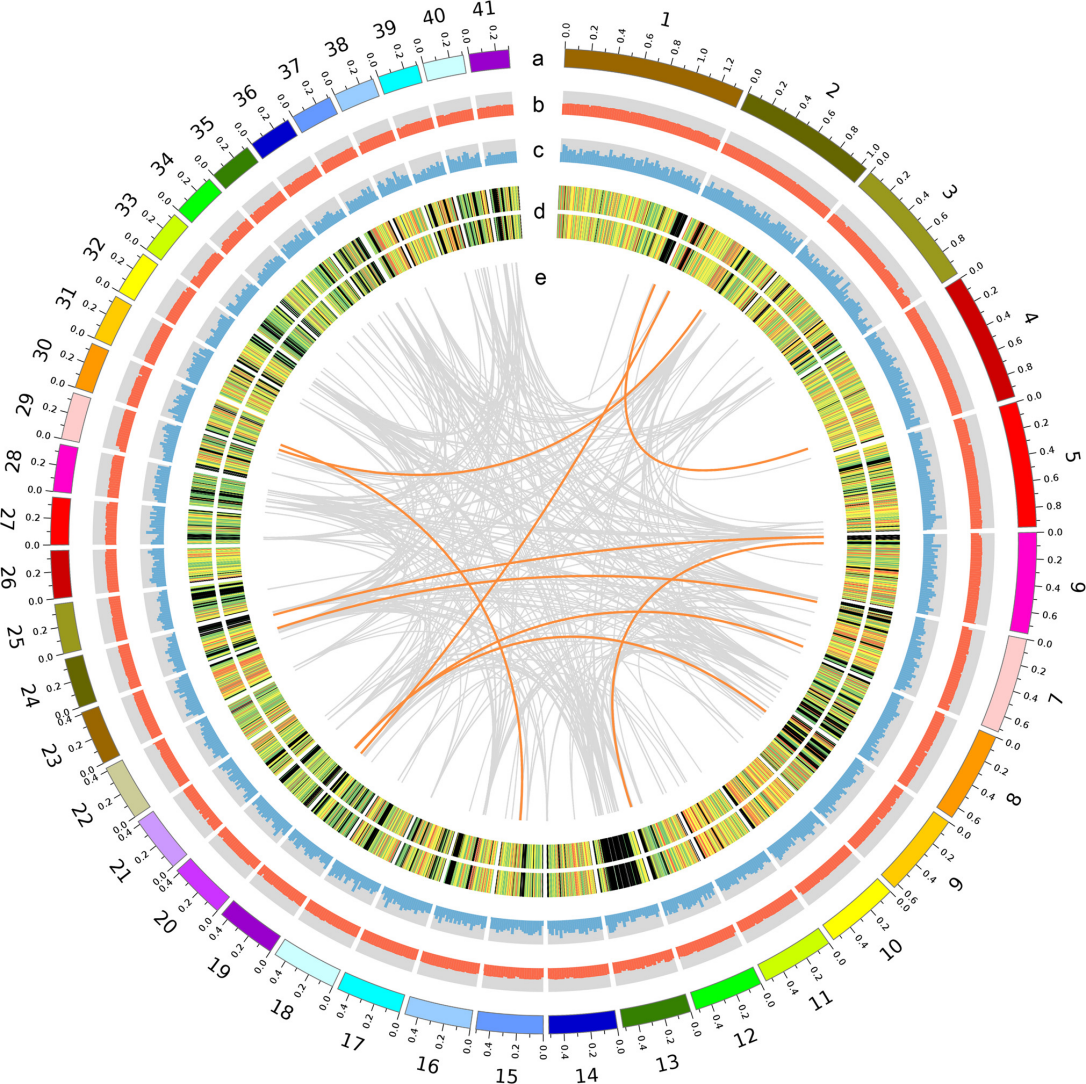
The ideogram showing the genomic features of *Lentinula edodes*. (a) Scaffolds: the diagram represents 41 scaffolds of *L*. *edodes*, half of the genome size. (b) GC content was calculated as the percentage of G+C in 20-kb non-overlapping windows. (c) Gene number was calculated in 20-kb non-overlapping windows, and the maximum value of the axis is 15. (d) Gene expression of 2 samples with red (FPKM > = 100), orange (FPKM > = 10), green (FPKM > = 0) and black (FPKM = 0) colors. The out ring presents the gene expression of mycelia cultured by medium with cellulose as the main carbon source, and the inner ring presents the gene expression of mycelia cultured by medium with glucose as the main carbon source. (e) Large segmental duplications: regions sharing more than 90% sequence similarity are connected by orange (sequence length > = 5kb) and grey (sequence length > = 2kb) lines.

The genome of monokaryotic *L*. *edodes* strain W1-26 (S1 Fig) was sequenced using a whole-genome shotgun sequencing strategy on the Illumina Hiseq 2000 platform. A 41.8Mb genome sequence was obtained by assembling approximately 97 million clean reads (~230 X coverage) (Table 1 and S1 Table). This genome sequence assembly consisted of 340 scaffolds with an L50 length of 300.7 kb and N50 of 41 (Fig 1 and S2 Table). Although unable to assemble these scaffolds into chromosomes, we estimated the genome size to be 48.3 Mb by k-mer analysis, with the scaffolds accounting for about 86.54% of the whole genome. In total, 14,889 gene models were predicted, with 35.82% undergoing alternative splicing (S3 Table). Repetitive sequences represent approximately 16.24% of the genome. The majority of the repeats were LTR/Gypsy (7.32% of the genome; S4 Table). Approximately 84% of the genes were annotated in similarity searches against homologous sequences and protein domains (S5 Table, S2 Fig and S3 Fig).

**Table 1:** General features of the *Lentinula edodes* genome.

Number of Scaffolds	340
Length of the genome assembly (Mb)	41.8
Scaffold L50^a^ (kb)	300.7
Contig L50^a^ (kb)	106.4
GC content (%)	46.1
Number of protein-coding genes	14,889
Average / Median gene length (bp)	2,217 / 1,889
Average / Median coding sequence size (bp)	1,440 / 1,146
GC content of protein-coding sequences (%)	48.6
Average / Median number of exons per gene	6.4 / 5
Average / Median exon size (bp)	286 / 162
Average / Median intron size (bp)	70 / 56
Average / Median size of intergenic regions (bp)	716 / 275

^a^ L50, length of the shortest scaffold or contig among those that collectively covered 50% of the assembly.

### Comparisons with other fungal genomes

**Fig 2:**
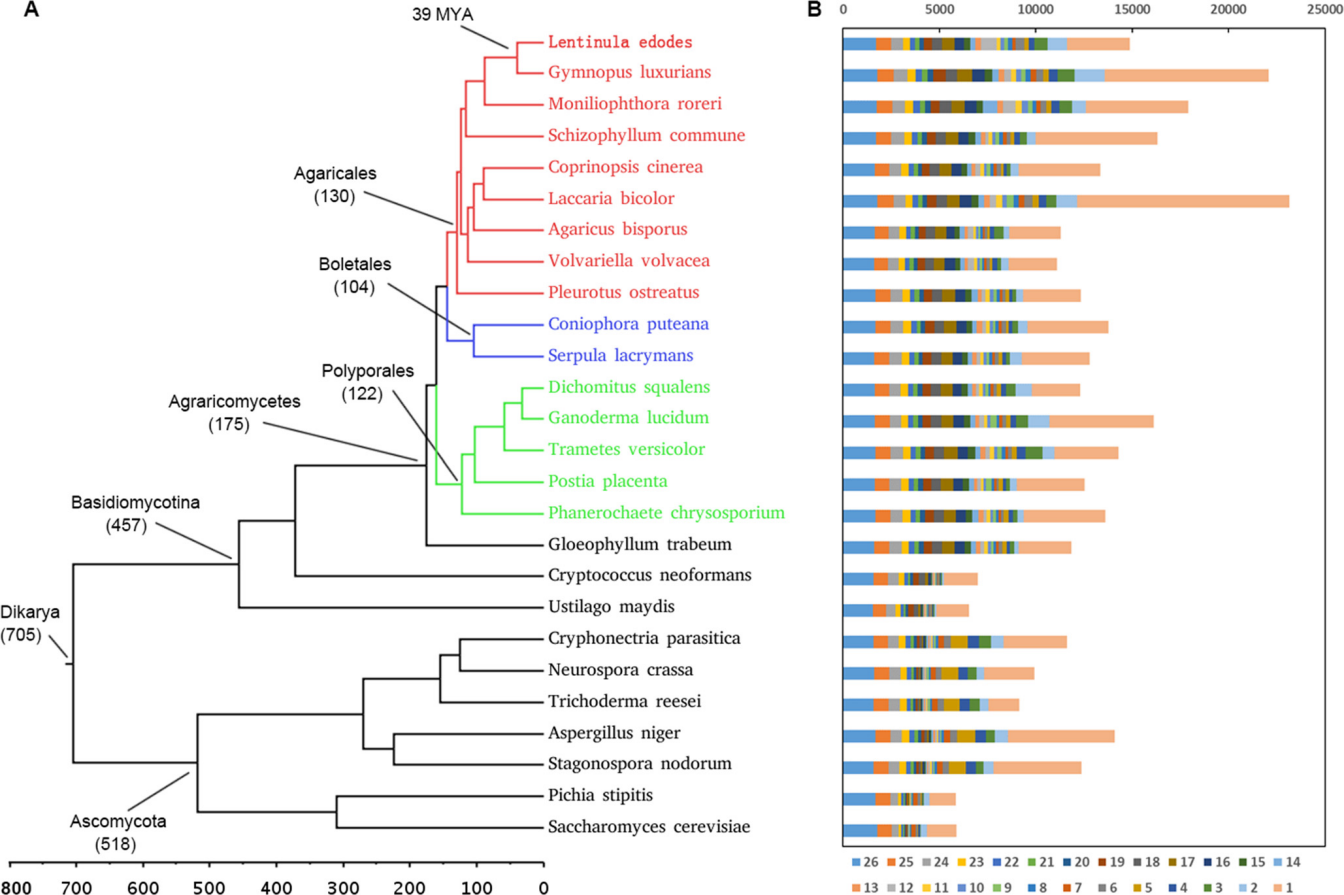
Orthologous gene number and phylogenetic tree of *Lentinula edodes* with other 25 fungal species. (a) The topology of the phylogenetic tree was constructed by the maximum likelihood method (bootstrap = 1000, LG+I+G+F model), and all bootstrap values were 100%. Time scale was shown by MYA (million years ago). (b) Orthologous gene number was calculated in each fugal species at 26 different levels.

The predicted proteome of *L*.*edodes* was compared with 25 other sequenced fungi (S6 Table). OrthoMCL analysis showed that a total of 25,344 Ortholog Cluster Groups (OCGs) were constructed, and among them, 8,578 OCGs contained 12,968 *L*. *edodes* proteins. About 11.5% of the predicted proteins in *L*. *edodes* had orthologs in all the other species, whereas 21.9% of the proteins were unique to *L*. *edodes*, approximately 41.1% of which had at least one paralog (Fig 2 and S7 Table). To illuminate the evolutionary history of *L*. *edodes*, a phylogenetic tree was constructed using 756 single-copy orthologous genes conserved in these 26 fungi (Fig 2 and S4 Fig). The topology of the tree was consistent with the taxonomic classification of these species. Meanwhile, molecular clock analysis revealed that *Gymnopus luxurians* [24] had the closest evolutionary affinity with *L*. *edodes*, and their divergence time was estimated to be 39 million years ago (MYA).

A total of 336 syntenic blocks were identified on the basis of the conserved gene order between *L*. *edodes* and *G*. *luxurians*, corresponding to 5,286 genes and 5,295 genes in each genome, respectively. On average, each block in the *L*. *edodes* genome included 16 genes in each of them. In total, 185 blocks contained more than ten genes. It is worth noting that, according to the largest block which contains 320 genes, the longest scaffold of *L*. *edodes* can be totally mapped to the longest scaffold of *G*. *luxurians* (S5 Fig).

The evolution and expansion of 7,527 OCGs with a family size of at least 13 in all 26 fungal species were examined using CAFE [25], and 312 / 689 OCGs were found to have undergone expansion / contraction in *L*. *edodes*. 42 expanded OCGs of *L*. *edodes* contained at least 10 genes (S8 Table), and the largest expanded OCG had 171 genes in *L*. *edodes*, but with unknown function. Interestingly, 10 OCGs were Retrovirus-related Pol polyproteins or transposon polyproteins and 1 OCG was Probable RNA-directed DNA polymerase from transposon X-element. Ribonuclease, ATP-dependent DNA helicase, chromobox protein, E3 ubiquitin-protein ligase and Ankyrin repeat domain-containing protein were also expanded in *L*. *edodes*. However, 21 OCGs could not be annotated by Swiss-Prot.

### Analysis of the matA and matB gene loci

**Fig 3:**
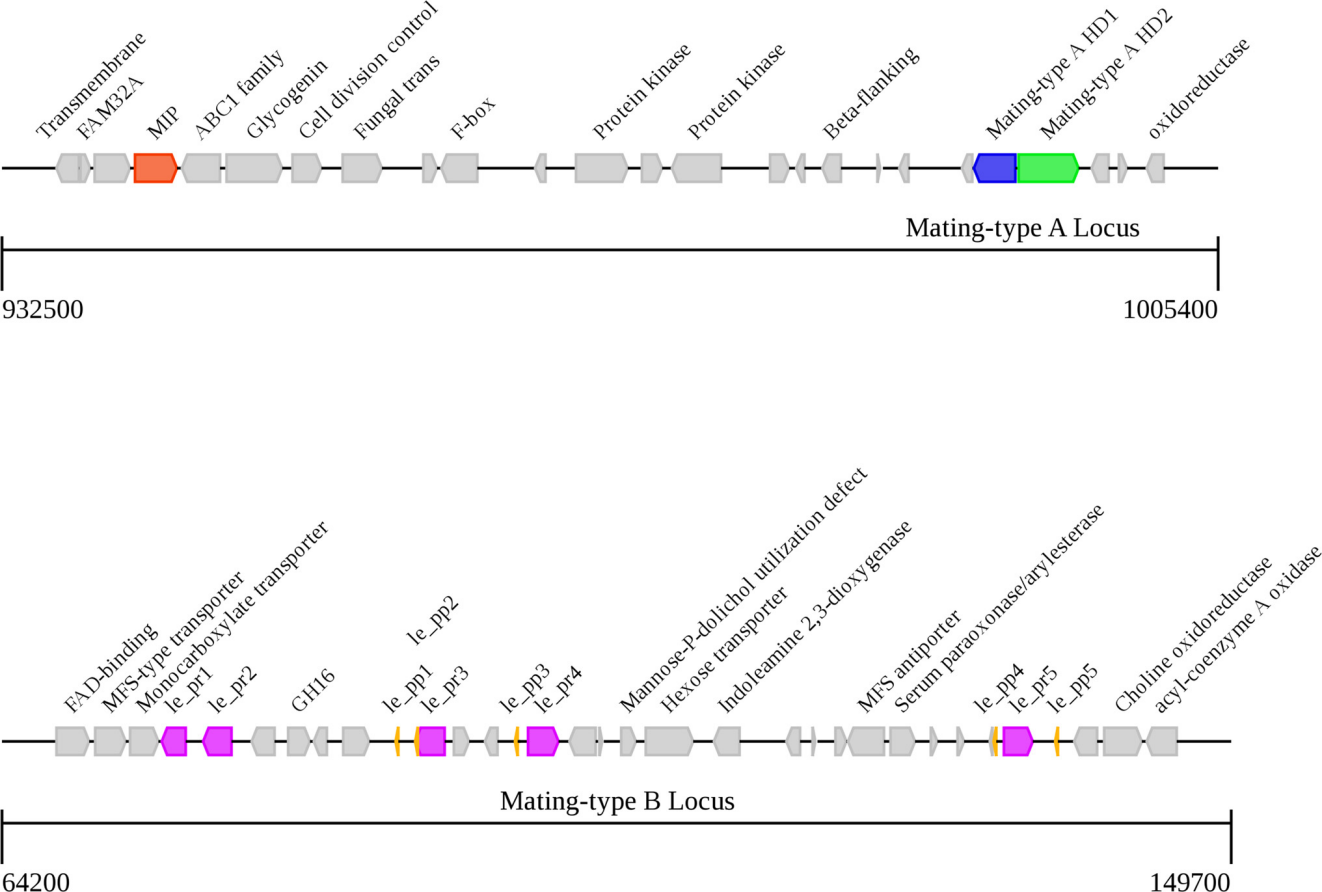
Distribution of genes in the matA and matB loci of *Lentinula edodes* strain W1-26. The matA and matB loci are positioned on scaffold 1 and 53, respectively. We identified 3 additional pheromone receptor like genes on scaffold 6, 28, and 67.

Two unlinked mating type loci, A and B, were identified from the genome sequence of *L*. *edodes* (Fig 3 and S9 Table). The typical A-mating-type locus, including two intact genes for HD1 and HD2 homeodomain transcription factors, is located on scaffold 1. Additionally, the mitochondrial intermediate peptidase (MIP) gene is located on the same scaffold as the A locus and the physical distance between these two loci was about 47.8 kb, which is consistent with the previous report in strain L54A [26]. In terms of B mating type genes located on scaffold 53, a total of five pheromone receptors and five pheromone precursor genes were identified, which were more than the pheromone receptor and precursor genes found from monokaryotic strains 939P42 and 939P26, respectively [27]. The varied numbers of the pheromone receptors and pheromone precursors may have originated from the differentiation of the *L*. *edodes* strains. Furthermore, three pheromone receptor-like genes were identified in the other three scaffolds but without pheromone genes in flanking 20 kb region, which was in accordance with the previous studies [28,29].

### Genes related to the unique aroma of *Lentinula edodes*

The unique aroma of *L*. *edodes* can be significantly emitted when drying the fruiting body at 55–60°C. Lenthionine (1,2,3,5,6-pentathiepaneis), belonging to an organosulfur compound, was identified as the primary volatile flavor of *L*. *edodes* [30]. Lenthionine has been reported to be generated from lentinic acid (a γ-L-glutamyl-cysteine sulfoxide precursor) and two enzymes are involved in the formation of lenthionine [31]. These two enzymes are Gamma-glutamyl transpeptidase and C-S lyase, which are encoded by 7 *ggt* genes and 5 *Csl* genes in the genome of *L*. *edodes*, respectively.

Gamma-glutamyl transpeptidases (GGTs; EC 2.3.2.2) which are involved in glutathione metabolism and in the cell defense mechanism against oxidative stress have been cloned from various species such as bacteria or mammals [32]. Among the 26 fungi, generally 2–4 genes encoded GGT except for *L*. *edodes* which is encoded by 7 *ggt* genes (S10 Table) as identified by Swiss-Prot annotation. More *ggt* genes may suggest the higher ability of catalyzing lentinic acid to L-cysteine sulfoxide derivative in *L*. *edodes* so that more organosulfur compounds can be synthesized.

C-S lyase encoded by the gene of LE01Gene02830 in the genome of *L*. *edodes* has been found to be a novel cysteine desulfurase (EC 2.8.1.7) which has cysteine sulfoxide lyase (EC 4.4.1.4) activity [33]. Through the similarity analysis with gene LE01Gene02830 by BLASTP, a total of 5 genes were identified to be C-S lyase genes in the genome of *L*. *edodes*. When compared with 1 or 2 genes encoding cysteine desulfurase in *A*. *bisporus* [17], *Coprinus cinereus* [34] or *Laccaria bicolor* [28], *L*. *edodes* and *G*. *luxurians* are found to have more C-S lyase genes, while no C-S lyase exists in *P*. *ostreatus* [35] or *V*. *volvacea* [20,36]. This observation may suggest the potential of *L*. *edodes* in producing more aroma and formaldehyde than other edible fungi.

### CAZymes and Genes involved in Lignocellulose decomposition

**Fig 4:**
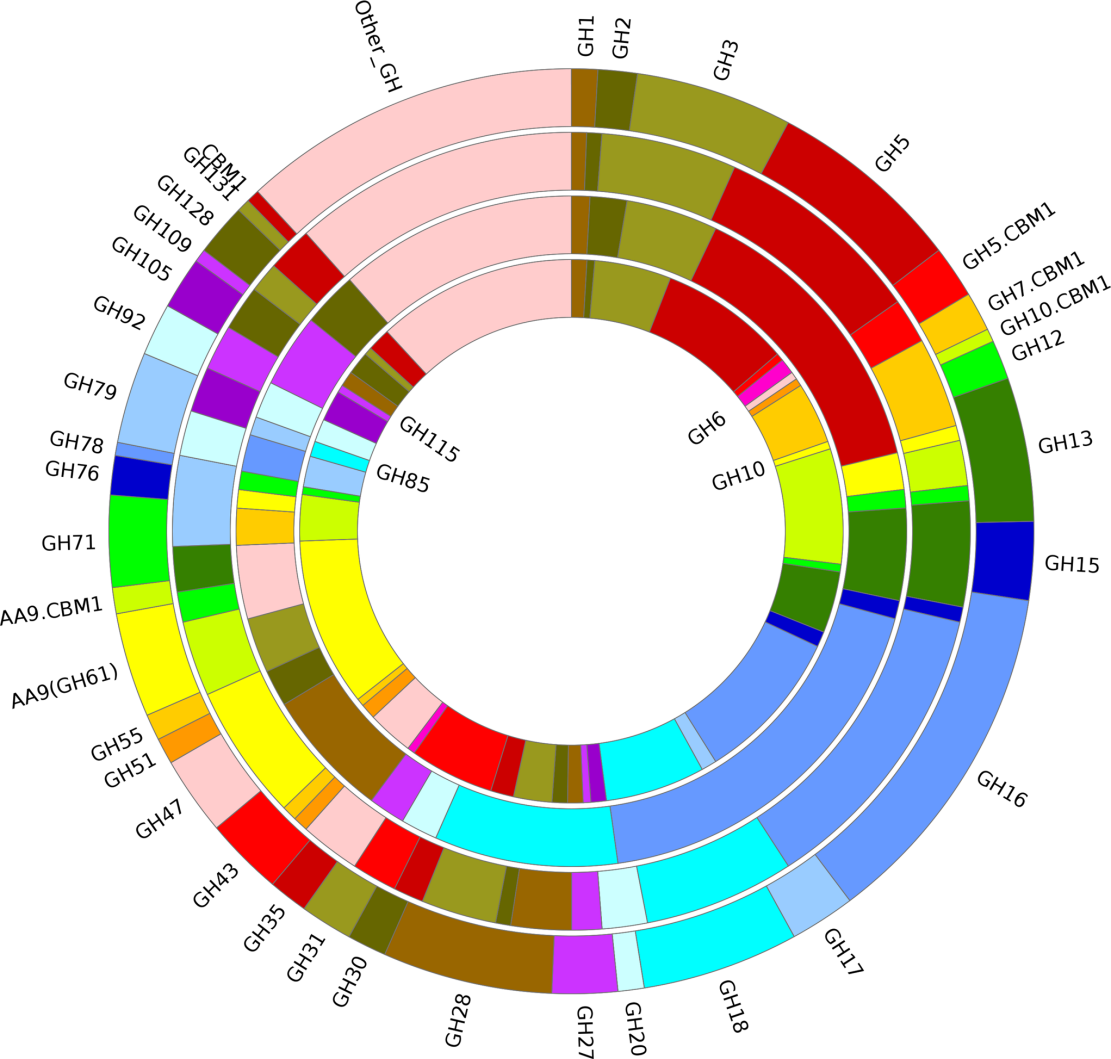
Distribution of various glycoside hydrolases and carbohydrate binding module 1. *Lentinula edodes* (out ring), *Phanerochaete chrysosporium* (second ring counted from out), *Postia placenta* (third ring) and *Volvariella volvacea* (inner ring).

A total of 461 candidate carbohydrate-active enzyme genes (CAZymes) were identified in the genome of *L*. *edodes*, which included 245 glycoside hydrolases, 31 carbohydrate esterases, 75 glycosyl transferases, 9 polysaccharide lyases, 58 carbohydrate-binding modules and 85 Auxiliary Activities enzymes (S11 Table). These CAZyme genes were identified by using our own pipeline with the combination of the HMM and BLASTP search methods. Compared to the genomes of other edible fungi, *L*. *edodes* has the highest number of glycoside hydrolases and glycosyl transferases. Additionally, the genome of *L*. *edodes* is particularly rich in members of the glycoside hydrolase families of GH13, GH15, GH17, GH27, GH71 and the carbohydrate-binding module family CBM20 (Fig 4 and S11 Table), indicating that *L*. *edodes* has a high potential of starch degradation. Many GH family genes which were predicted as cellulases or hemicellulases were also annotated to belong to carbohydrate-binding module family CBM1, such as GH5, GH7, GH10 and AA9 (formerly GH61) (Fig 4). The CBM1 has the cellulose-binding function, suggesting that these genes containing CBM1 may play a more important role in cellulose degradation.

Additionally, 38 candidate cellulase genes were identified in the genome of *L*. *edodes*, which were similar to other white rot fungi such as *G*. *lucidum* [18] and *Phanerochaete chrysosporium* [37], but less than the straw-rotting mushrooms of *C*. *cinerea* [34] and *V*. *volvacea* [21]. The brown rot fungi *Postia placenta* [38] and *Serpula lacrymans* [39] have the lowest number of cellulase genes despite their high cellulose depolymerization efficiency (Table 2 and S12 Table). In the same way, 9 putative hemicellulase and 7 putative pectinase encoding genes were identified in the genome of *L*. *edodes*, which were also less in number than the genes number in straw-rot mushrooms but higher than that in brown rot fungi. *L*. *bicolor*, which belongs to ectomycorrhizal fungi, and *A*. *bisporus*, which usually grows on secondary fementation materials have the lowest number of genes encoding cellulase, hemicellulase and pectinase.

**Table 2:** The distribution of lignocellulolytic genes in *Lentinula edodes* and other edible fungi or model fungi.

Class	Enzyme	EC	CAZy	*L*. *edo*	*S*. *com*	*G*. *luc*	*P*. *chr*	*P*. *ost*	*P*. *pla*	*S*. *lac*	*C*. *cin*	*V*. *vol*	*L*. *bic*	*A*. *bis*
				White rot					Brown rot		Straw rot			
**Cellulase**	Endo-beta-1,4-glucanase	EC:3.2.1.4	GH5, GH7, GH9, GH12, GH44, GH45, AA9	21	24	21	19	34	7	12	36	26	9	15
	1,4-β-cellobiosidase	EC:3.2.1.91	GH6, GH7	5	3	4	8	16	0	1	10	16	0	2
	β-glucosidase	EC:3.2.1.21	GH1, GH3	12	12	10	10	11	6	11	9	12	2	6
	Total	-	-	38	39	35	37	61	13	24	55	54	11	23
**Hemicellulase**	Endo-1,4-beta-xylanase	EC:3.2.1.8	GH10, GH11	5	6	7	7	5	3	1	12	16	0	4
	β-xylosidase	EC:3.2.1.37	GH3, GH39,GH43	2	4	4	1	5	1	1	1	3	0	2
	α-glucuronidase	EC:3.2.1.131	GH67	0	0	0	0	0	0	0	0	0	0	0
	acetylxylan esterase	EC:3.1.1.72	CE1, CE5	1	4	2	4	1	0	0	3	2	1	3
	feruloyl esterase	EC:3.1.1.73	CE1	0	6	0	0	1	0	0	2	2	0	0
	α-L-arabinofuranosidases	EC:3.2.1.55	GH51, GH54, GH62	1	1	1	1	1	0	0	0	0	0	0
	Total	-	-	9	21	14	13	13	4	2	18	23	1	9
**Pectinase**	pectin lyase	EC:4.2.2.10	PL1	0	0	0	0	0	0	0	0	0	0	0
	pectate lyase	EC:4.2.2.2	PL1, PL3, PL9	3	10	0	0	12	0	0	3	14	0	3
	pectinesterase	EC:3.1.1.11	CE8	2	2	3	2	2	2	2	0	3	4	2
	polygalacturonase	EC:3.2.1.15	GH28	2	0	1	1	1	2	1	0	0	0	1
	Total	-	-	7	12	4	3	15	4	3	3	17	4	6
**Lignin Oxidase**	multicopper oxidase	EC:1.10.3.2	AA1	14	3	15	3	12	4	6	16	11	12	13
	Lignin peroxidase	EC:1.11.1.14	AA2	0	0	2	10	0	0	0	0	0	0	0
	Manganese peroxidase	EC:1.11.1.13	AA2	2	0	2	5	0	0	0	0	2	0	0
	Versatile peroxidase	EC:1.11.1.16	AA2	1	0	5	1	9	0	0	0	5	1	2
	Other peroxidase	-	AA2	6	2	1	1	1	2	1	4	3	2	3
	Total	-	-	23	5	25	20	22	6	7	21	21	15	18
**Lignin Degrading Auxiliary Enzyme**	aryl-alcohol oxidase	EC:1.1.3.7	AA3_2a	8	0	7	0	0	0	0	27	0	4	22
	glucose oxidase	EC:1.1.3.4	AA3_2b	2	7	3	7	2	3	3	1	1	1	0
	alcohol oxidase	EC:1.1.3.13	AA3_3	4	4	4	3	4	5	5	2	5	2	5
	Pyranose oxidase	EC:1.1.3.10	AA3_4	1	1	0	1	0	0	0	0	0	0	0
	vanillyl-alcohol oxidase	EC:1.1.3.38	AA4	2	3	2	2	2	2	3	0	2	1	1
	Glyoxal oxidase	EC:1.1.3.-	AA5_1	3	0	8	6	8	1	2	5	2	5	9
	Galactose oxidase	EC:1.1.3.9	AA5_2	2	2	1	1	7	1	1	1	2	5	0
	Benzoquinone reductase	EC:1.6.5.6	AA6	2	4	1	4	2	1	2	3	2	2	4
	Total	-	-	24	21	26	24	25	13	16	39	14	20	41
**Total**	-	-	-	101	98	104	97	136	40	52	135	129	51	97

As a typical white rot fungus, *L*. *edodes* can degrade all plant cell wall components, and has a particularly high lignin degradation efficiency [8]. Lignin peroxidases (LiPs), manganese peroxidase (MnPs) and versatile peroxidases (VPs) are the main enzymes for lignin decomposition. Two candidate MnPs and one candidate VP genes were identified in the genome of *L*. *edodes* and LiP gene was absent (Table 2). In addition, 14 putative multicopper oxidases encoding genes including laccases were also identified, 3 more than the number previously reported [21]. Furthermore, 6 genes encoding other candidate peroxidases such as L-ascorbate peroxidase and 24 genes encoding lignin degrading auxiliary enzymes were identified and they may participate in the lignin decomposition.

In summary, the genome of *L*. *edodes* revealed that 101 gene models (S13 Table) were potentially involved in lignocellulose decomposition, with similar composition to the model of white rot fungi.

### Transcription factors and secondary metabolites

A total of 474 transcription factor genes were identified in the genome of *L*. *edodes*, and most of these genes belong to zf clus, zf-C2H2 or Fungal_trans (S15 Table). 32 secondary metabolite gene clusters were identified and most of them are involved in the synthesis of terpene, T1pks and bacteriocin (S16 Table).

### RNA-Seq and gene expression analysis

We used RNA-seq of Illumina Hiseq 2000 platform to compare the whole-genome expression when the mycelia of *L*. *edodes* were cultured with glucose or cellulose as main carbon source. Of the 14,889 predicted genes, 10,629 (71.4%) were expressed in at least one sample with the cutoff FPKM value of 1, and the expression of these genes are useful for the genome annotation (S16 Table). With the FDR value of 0.001 and |log2(fold-change)| value of 1.0 as cutoffs, 317 genes were up-regulated and 336 genes were down-regulated with the mycelia of *L*. *edodes* cultured in cellulose medium versus glucose medium (S17 Table). Among the 4 differentially expressed cellulase genes, two (LE01Gene08227 and LE01Gene09249) were up-regulated and two (LE01Gene08136 and LE01Gene13984) were down-regulated in cellulose medium (Table 3 and S18 Table). The number of differentially expressed cellulase genes is less than our expectation. However, the expressions of 23 CAZyme genes were up-regulated in the cellulose medium, suggesting the existence of a significant difference in the patterns of carbon source utilization between cellulose medium and glucose medium (*p* = 1.4e-4 by Fisher’s exact test through 4 values: 23, 461, 317 and 14889). The expression of these genes may have been affected by the transcription factor (TF) genes (*p* = 4.7e-2 by Fisher’s exact test through 4 values: 16, 474, 317 and 14889), for 16 TF genes were up-regulated in the cellulose medium. Interestingly, the median FPKM value of cellulose degrading genes was 3.87, less than that of CAZyme genes (7.53) (Table 3), implying that the mycelia of *L*. *edodes* at that stage may have a low ability of cellulose degradation.

**Table 3:** RNA-Seq data of CAZyme genes and transcription factor genes of *L*. *edodes*.

Gene Type	Gene number	Up-regulated DEG number	Down-regulated DEG number	Median / Mean FPKM (Glucose medium)	Median / Mean FPKM (Cellulose medium)
Cellulose degrading gene	38	2	2	3.87 / 16.4	3.6 / 16.9
Lignocellulose degrading gene	101	5	3	3.97 / 29.5	3.99 / 32.5
CAZyme gene	461	23	12	7.53 / 49.1	8.82 / 50.2
Transcription factor gene	474	16	6	7.99 / 27.3	8.99 / 29.2
All genome gene	14889	317	336	6.17 / 62.6	6.38 / 55.4

Note: The CAZyme genes contain all lignocellulose degrading genes, and the lignocellulose degrading genes contain all cellulose degrading genes.

## Discussion

*L*. *edodes* is one of the most popular, edible cultivated mushroom species and it is also an important fungus in cellulose and lignin degradation with potential for bioenergy production. In the present research, we chose the strain W1 for genome sequencing because it is suitable for artificial cultivation. The genome sequences of its haploid spore strain W1-26 were analyzed using the Illumina Hiseq 2000 platform. We assembled the genome into 340 scaffolds with a size of 41.8 Mb which is less than the estimated genome size of 48.3 Mb, indicating that 6.5 Mb (13.5%) genome sequence cannot be assembled. In fact, the sequences that failed to be assembled were likely to be the repetitive sequences, and similar situations were also reported in the other strains of *L*. *edodes* [22,40]. This suggests that the genome sequences of *L*. *edodes* are hard to be sequenced and assembled, which is probably related to the high expansion of retro-transposon gene families and the high percentage of repetitive sequences. The third generation sequencing technology can be more efficient in solving the high repeat ratio problem. Recently, PacBio RS II and Illumina Hiseq 2500 platforms have been jointly used for genome sequencing of *L*. *edodes* monokayon B17, and 46.1 Mb genome sequences consisting of 31 scaffolds were assembled [41]. This genome is much more complete and less fragmented due to the application of the third generation sequencing technology.

The matA and matB loci regulate the fusion of different monokaryon mycelia and the formation of the fertile dikaryon. According to a genetic linkage map containing 86 insertion-deletion (InDel) molecular markers (unpublished data), the InDel marker S278 proximate to matA located at 905,409 bp position on scaffold 1 is just close to the matA genes. Similarly, the InDel marker S323 proximate to matB located at 70,919 bp position on scaffold 53 is merely close to the matB genes. In addition, 79 InDel markers can be mapped to 55 scaffold sequences which represent 44.4% (18.59 Mb) of the genome size. This indicated the high accuracy of our genome sequences and genetic linkage map.

The unique aroma of *L*. *edodes* is an important factor for its high popularity with consumers, and the compounds of the flavor are mainly lenthionine [30]. In the genome of *L*. *edodes*, 7 genes encoding candidate Gamma-glutamyl transpeptidases and 5 genes encoding candidate C-S lyases are involved in the pathway from lentinic acid to lenthionine. However, the synthetic pathway of lentinic acid and the transformation mechanism from thiosulfinate to lenthionine are still unknown. *L*. *edodes* has the highest number of genes encoding GGTs and C-S lyases among the edible mushrooms we examined, which may explain why the flavor of *L*. *edodes* is special compared with other edible mushrooms.

The comparative analysis of genes related to lignocellulose degradation in *L*. *edodes* and other edible mushrooms or model fungi reveals that *L*. *edodes* has a similar component of gene families to that of other white rot fungi such as *G*. *lucidum* and *P*. *chrysosporium* except for *P*. *ostreatus*. Interestingly, *P*. *ostreatus* and straw-degrading fungi *C*. *cinerea* and *V*. *volvacea*, have a larger number of cellulase genes.

The comparative transcriptome analysis identified only 2 cellulase genes which were up-regulated after 120 minutes of cultivation in the cellulose medium. These 2 genes may be the key genes for cellulose degradation, and their potential interaction with 16 up-regulated transcription factor genes would be meaningful for the research of cellulose degradation. From these findings, it can be seen that the FPKM values of lignocellulolytic genes are lower than those of most genes, probably because 120 minutes were too short to induce their expression, and more time was needed to increase the expression of the lignocellulolytic genes in the cellulose medium. A previous study reported that the expression of 356 genes of *Phanerochaete chrysosporium*, including some lignin peroxidases, manganese peroxidases, and auxiliary enzymes, accumulated to relatively high levels at 96 h, which was at least four times the levels found at 40 h after inoculation with solid spruce wood [42].

*L*. *edodes* is widely cultivated in China with the classic substance formula (78% hard wood shaving, 20% wheat bran, 1% gypsum and 1% sugar, natural dry weight), but the application of this formula will consume an excessive amount of sawdust, leading to large deforestation. According to the statistics issued by China Edible Fungi Association, the yield of *L*. *edodes* in China in 2013 was 7.10 million tons (http://www.cefa.org.cn/2014/12/15/8002.html), suggesting that about 5.83 millon tons of dry timber would be used every year with the normal biological conversion efficiency of *L*. *edodes* being 1 kilogram dry medium substance for the production of 0.95 kilogram of fresh shiitake mushroom fruiting body. However, agricultural straws are possible alternatives to sawdust. Recently, wheat straw has been utilized in *L*. *edodes* cultivation, and *L*. *edodes* seems to have fairly good biological efficiency and higher degradation ability to lignin and hemicellulose than cellulose, but the yield and quality are not as high as those of sawdust [43,44]. Although sawdust has been required in the cultivation of *L*. *edodes* currently, part of sawdust can be expected to be replaced with various straws in the cultivation materials to obtain high yield and high quality. This research provides insights into the lignocellulolytic genes of *L*. *edodes*, and thus facilitates our understanding of the transforming process of the substance during the cultivation of *L*. *edodes*.

## Materials and Methods

### Strains and culture conditions

*Lentinula edodes* monokaryotic strain W1-26 which germinated from one of the spores of dikaryotic strain W1 (ACCC50926) was used for whole genome sequencing, and *L*. *edodes* strain W1 was used for RNA-Seq. Vegetative mycelia of W1-26 were cultivated by Potato Dextrose liquid medium in the dark at 26°C for about 12 days, and then were collected for genome sequencing. Similarly, the vegetative mycelia of W1 were cultivated by CYM liquid medium for about 20 days until they occupied the entire cultivation space, and then the mycelia were collected, washed by sterile water, and transferred to 2 different mediums. The first glucose medium is normal CYM liquid medium with extra 2% glucose and 1‰ sodium lignin sulfonate. The other cellulose medium is CYM liquid medium with 2% cellulose and 1‰ sodium lignin sulfonate without the addition of 2% glucose. After 120 minutes of cultivation, mycelial samples in the two mediums were collected separately for strand specific RNA-seq experiments, and each experiment was performed in triplicate biologically.

### DNA sequencing and data preprocessing

About 100 μg of genomic DNA samples were used for genome sequencing on the Illumina Hiseq 2000 platform by Novogene Biotech AG (Beijing, China). Two paired-end libraries (170bp, 440bp) and 3 mate-paired libraries (2300bp, 4800bp, 5000bp) with different insert sizes were constructed and a total of 178.66M raw reads were produced.

The raw data were preprocessed by the following steps. Firstly, reads were aligned to the adapter sequences, which were truncated according to alignments. Secondly, NGS QC Toolkit [45] was used to filter low quality reads by satisfying one of these three conditions: bases with quality < = 20 were regarded as low quality bases, and the percentage of low quality bases in a read > = 40%; or ambiguous bases’ percentage of a read > = 10%; or the read length < 50bp. Thirdly, FastUniq [46] was used to remove the PCR duplicates. Finally, reads were aligned to *L*. *edodes* mitochondrial genome sequence by Bowtie2 [47], and the read pairs, which failed to match the mitochondrial genome sequence, were picked out. After all the aforementioned steps, clean reads were produced.

### Genome survey and assembly

The clean data of the 2 paired-end libraries were inputted to software GCE [48] for genome survey. The genome size was estimated to be 48.3 Mb, and the percentage of the sequences repeated at least twice in the genome was 31.87%. In addition, the genome survey information was also obtained by the FindErrors module when ALLPATHS-LG [49] was used to assemble the genome sequences. According to the log information of ALLPATHS-LG, the genome size was estimated to be 48.1 Mb and 29.3% of the genome size was estimated to be repetitive at least twice. The genome survey results from these two methods were similar. According to the information, the Illumina library preparation strategy and sequencing data size were determined for whole-genome *de novo* sequencing.

ALLPATHS-LG assembler software produced a genome with a size of 41,822,111 bp and a scaffold L50 of 237,901 bp. Then, the mate-paired library data and transcript sequences assembled by Inchworm module of Trinity with default parameters were inputted to ABySS [50] for re-scaffolding the ALLPATHS-LG assembly. The genome scaffold L50 was improved to 296,016 bp. Next, ICORN2 [51] was used to correct SNPs and InDels, and GapFiller [52] was used to close gaps. Finally, SOPRA [53] was used for re-scaffolding again, and ICORN2 and GapFiller were used in turn to obtain the final genome assembly.

### Repeat, rRNA, and tRNA identification

RepeatMasker and RepeatModeler (http://repeatmasker.org) were used to detect and annotate transposable elements, satellites, simple repeats and low-complexity sequences. rRNAs were identified by RNAmmer [54] and Rfam [55]. tRNAscan-SE [56] was used to detect tRNA regions and its secondary structures.

### Protein-coding gene prediction and functional annotation

The transcript sequences were assembled by Trinity using RNA-Seq data. Then, the inchworm sequences with length > = 150 bp were inputted to PASA [57,58], and 3582 complete gene models were derived. According to these gene models, AUGUSTUS [59] and SNAP [60] HMM parameters were trained.FMAKER [61] was used to predict the gene models with these input data: repeat database created by RepeatModeler; transcript sequences assembled by PASA; HMM files of AUGUSTUS, SNAP and GeneMark-ES; fungal protein sequences derived from NCBI protein database by searching “(txid5338[Organism:exp]) NOT partial”. Of the 9,641 gene models predicted by MAKER, 6,980 gene models with AED value < = 0.1 were picked out for AUGUSTUTS and SNAP training again. The sensitivity of precision by AUGUSTUS is 0.583 at the gene level. Meanwhile, the UTR HMM parameters were also trained by AUGUSTUS with CRF (Conditional Random Field). With more accurate HMM parameters of AUGUSTUS and SNAP, MAKER was used again to predict gene models with the option “keep_preds = 1”, and 12,676 gene models were predicted by MAKER.

At the same time, AUGUSTUS alone predicted 12,547 gene models with intron and exon hints as input data. The hints were created by the alignment of RNA-Seq data. The gene models of AUGUSTUS contain UTR and have more accurate boundary between intron and exon. Additionally, SNAP and GeneMark-ES [62] predicted 15,933 and 13,928 gene models, respectively. Then, according to the visualization of RNA-Seq data, WebApollo [63] was used to integrate and modify the gene models predicted by MAKER, AUGUSTUS, GeneMark-ES and SNAP one by one manually. Finally, 14,945 gene models were produced.

Alternative splicing (AS) was analyzed by SpliceGrapher [64]. Firstly, SpliceGrapher was used to identify alternative splicing events from the SAM format file produced by the alignment of RNA-Seq data. Then, all the AS transcripts were constructed and their expression values were calculated by SpliceGrapher. Next, the AS transcripts with FPKM > = 1 were inputted to PASA pipeline for a more accurate AS-affected gene identification and update of the gene models. The ultimate number of *Lentinula edodes*’ gene models is 14,889.

All of the predicted gene models were functionally annotated based on similarity to annotated genes. BLASTP [65] was used to align the protein sequences to Nr, Swiss-Prot [66], COG [67], and KOG [68] protein databases with e-value < 1e-5. The gene models were also annotated by their protein domains using InterPro database [69] and CDD database [70]. On the basis of Nr and InterPro databases, Blast2go [71] was used to classify all genes by Gene Ontology (GO). Additionally, KEGG annotation was taken by submitting genomic protein sequences to KAAS [72] with BBH (bi-directional hit) method.

### Species tree construction and gene family expansion analysis

Together with *L*.*edodes*, 26 fungal species assigned to Basidiomycota or Ascomycota were used in the phylogenetic analysis. The protein sequences of these 26 fungi were compared by BLASTP with e-value < 1e-5 and hit number < 500. Then, the BLASTP result was analyzed by OrthoMCL[73] with default parameters to get the orthologous genes, and 756 single-copy orthologous genes were determined. Multiple sequence alignments of these 756 genes were calculated by MAFFT v7.158b [74] software, and were combined into a long sequence for each species. Then, the conserved block regions of the alignment were picked out by Gblocks 0.91b with default parameters [75] of the software, and the final alignment length was 193323 aa. With the input of this alignment, phylogenetic tree was constructed by RAxML-8.0.26 [76] software with bootstrap 1000. Three fossil calibration points [77] were fixed in the molecule clock analysis: the most recent common ancestor (MRCA) of *Coprinopsis cinerea*, *Laccaria bicolor* and *Schizophyllum commune* were diverged at 122.74 MYA; the MRCA of *Serpula lacrymans* and *Coniophora puteana* were diverged at 104.23 MYA; the MRCA of *Pichia stipitis*, *Aspergillus niger*, *Cryphonectria parasitica*, *Stagonospora nodorum* and *Trichoderma reesei* were diverged at 517.55 MYA. Then, the divergence time of other nodes was calculated by r8s v1.80 [78] software with TN algorithm, PL method and the smoothing parameter value set to 1.8 through cross-validation. Based on the ultrametric tree, the orthologous gene family expansion was calculated by CAFE version 3 [25] software.

### Identification of matA and matB genes

The matA genes were identified by mapping genome protein sequences to the matA and MIP genes of *Coprinopsis cinerea* and *Schizophyllum commune*. The pheromone receptor genes were identified by the Swiss-Prot annotation with key word “Pheromone receptor”. The protein length of pheromone precursor is too short, usually 50~60 aa, so they could not be predicted in the normal genome annotation procedure. These genes were searched in ~20kb flanking sequence of the pheromone receptor genes by Transdecoder (https://transdecoder.github.io/) software with PFAM search. The ORFs annotated to PF08015.6 were pheromone precursor genes.

### Gene expression and differential expression analysis

The RNA-seq experiments were performed by Illumina Hiseq 2000 platform with standard Illumina reagent. Through the quality control by Trimmomatic [79] with parameters “ILLUMINACLIP:TruSeq3-PE.fa:2:30:10 LEADING:3 TRAILING:3 SLIDINGWINDOW:4:15 MINLEN:36”, each replicate of the 2 samples gained 12.5M clean read pairs averagely. Then, the RNA-seq reads of these 2 samples were aligned separately to genome sequences by HISAT2 [80] with the parameters “-min-intronlen 20—max-intronlen 4000—rna-strandness RF—score-min L,-0.3,-0.3”, resulting in an average of 69.6% aligment rate for all replicates. Then, the unique mapped alignments were extracted for the global genome expression calculation performed using cuffquant and cuffnorm [81]. Finally, the 2 samples, each with 3 biological replicates, were compared by cuffdiff [77] to obtain the differentially expressed genes with the cutoff threshold: FDR < = 0.001 and |log2Ratio| > = 1.

### Identification of CAZymes, Lignocellulolytic Genes and transcription factors

Carbohydrate-active enzymes (CAZymes) were classified separately by HMM search of dbCAN HMMs 4.0 [82] (default cutoff threshold) and BLASTP search of CAZy datebase [83] (evalue < = 1e-6 && covered fraction ratio > = 0.2, maximum hit number is 500). Then, according to the common results of these 2 methods, a series of more strict thresholds (BLASTP hit number and evalue, S19 Table) of each CAZyme family were determined by median values of 26 fungal genomes. Finally, the blastp results screened with the new threshold were added to the common results, to obtain the final CAZyme annotation. Therefore, the identification process used here is distinct from that employed by the CAZy system [83], suggesting the possibility of occasional discrepancies with previously published results. Lignocellulolytic Genes were identified mainly by the Swiss-Prot annotation with key words (S20 Table) among the CAZymes. Transcription factors were identified by a set of InterPro codes (S14 Table) which were collected according to TRANSFAC [84] and FTFD databases [85].

### Data availability and accession numbers

The genome sequences of *L*. *edodes* W1-26 have been deposited at GenBank under the accession number of LDAT00000000. Additionally, more data can be downloaded from our *Lentiula edodes* genome database website: http://LEgdb.chenlianfu.com. The version described in this paper is the first version. Apart from that, the genome sequencing reads have been deposited at GeneBank under the accession number of SRS875031, and RNA-Seq reads with the accession numbers of SRS1090734.

## Supporting Information

S1 Fig The monokaryotic and diploid mycelia of *L*. *edodes* under confocal laser scanning microscopic. (a,b,c) The haploid strain W1-26 has only one nucleus in each cell. (d,e,f) The diploid strain W1 has diploid nucleus in each cell. The 2 red arrows indicate the clamp connection and diploid nucleus.
(TIF)

S2 Fig KOG function classification of *L*. *edodes*’ genes. (TIF)

S3 Fig GO class of *L*. *edodes*’ genes. (TIF)

S4 Fig Phylogenetic analysis of 26 fungi based on 756 single copy orthologous genes. A maxlikehood phylogenetic tree of 26 fungal species was constructed using RAxML, and a bootstrap analysis with 1,000 replications was performed. All of the bootstrap values at any node were 100%.
(TIF)

S5 Fig The collinearity of *L*. *edodes* and *Gymnopus luxurians*. The two genome sequences shown in this picture stand for half of the genome size.
(TIF)

S1 Table Sequencing statistics. (DOCX)

S2 Table Assembly statistics. (DOCX)

S3 Table Gene model statistics. (DOCX)

S4 Table Classification of repeated sequences. (DOCX)

S5 Table Gene model supported by hits/data from the corresponding public databases. (DOCX)

S6 Table Resources of the other 25 fungi for OrthoMCL analysis. (DOCX)

S7 Table Orthologous gene classification of *L*. *edodes* and the other 25 fungi. (XLS)

S8 Table Expanded Gene families of *L*. *edodes*. (DOCX)

S9 Table The locus of matA and matB genes. (DOCX)

S10 Table Gamma-glutamyl transpeptidase genes of 26 fungi species. (DOCX)

S11 Table Gene distribution of CAZyme in *L*. *edodes* and the other 25 fungi. (XLS)

S12 Table The gene distribution of lignocellulolytic enzymes in *L*. *edodes* and other 25 fungi. (XLS)

S13 Table Lignocellulolytic genes of *L*. *edodes*. (DOCX)

S14 Table Transcription factor genes of *L*. *edodes*. (XLS)

S15 Table Secondary metabolite gene clusters of *L*. *edodes*. (XLS)

S16 Table Global gene expression of *L*. *edodes* (A: glucose medium; B: cellulose medium).
(XLS)

S17 Table Differentially expressed genes of *L*. *edodes* cultivated by Cellulose medium versus glucose medium. (XLS)

S18 Table Differentially expressed genes of *L*. *edodes*. (XLS)

S19 Table Thresholds of dbCAN blastp for each CAZyme family. (XLS)

S20 Table Key words for identification of the lignocellulolytic genes by Swiss-Prot annotation. (DOCX)
