# Supplementary figures and images for: Genome Sequence of the Edible Cultivated Mushroom Lentinula edodes (Shiitake) Reveals Insights into Lignocellulose Degradation
Source: PLoS One. 2016 Aug 8;11(8):e0160336. doi: 10.1371/journal.pone.0160336 (PMC4976891; doi:10.1371/journal.pone.0160336)

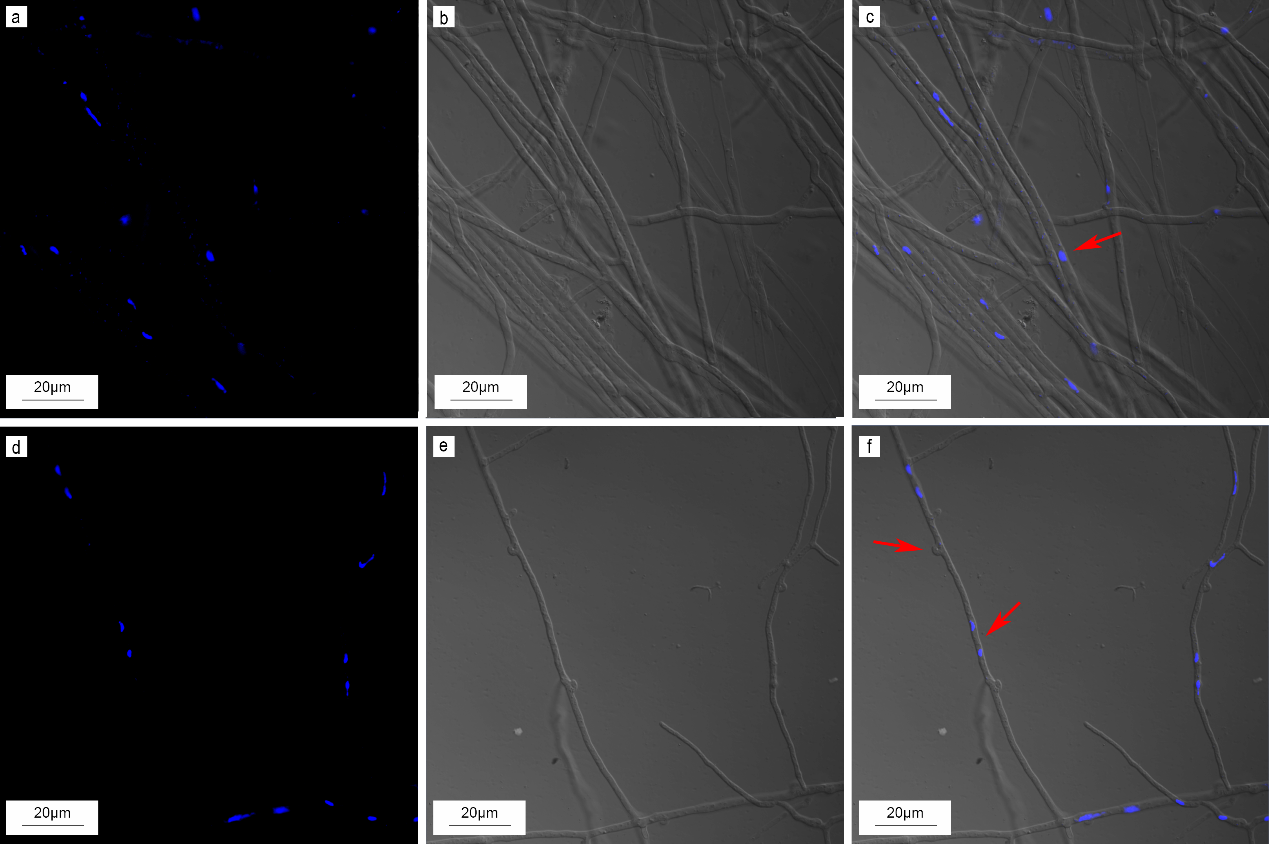

Supplement: S1 Fig — (a,b,c) The haploid strain W1-26 has only one nucleus in each cell. (d,e,f) The diploid strain W1 has diploid nucleus in each cell. The 2 red arrows indicate the clamp connection and diploid nucleus. (TIF) [file pone.0160336.s001.tif]

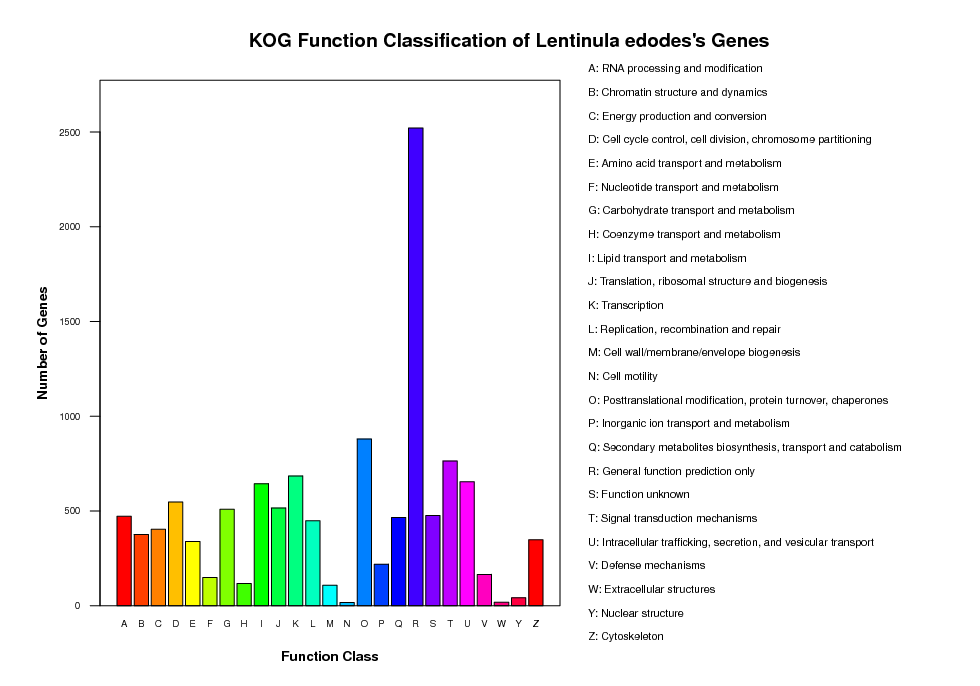

Supplement: S2 Fig — (TIF) [file pone.0160336.s002.tif]

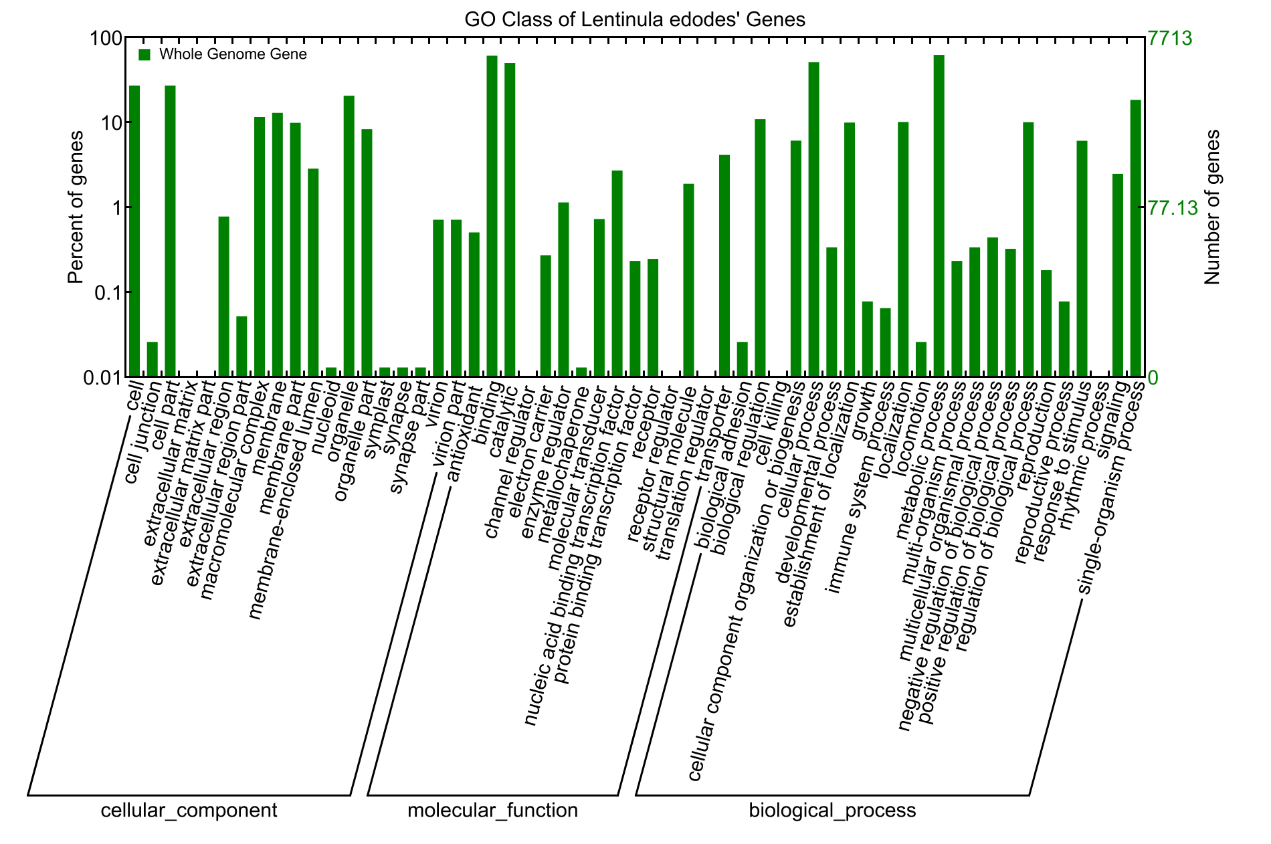

Supplement: S3 Fig — (TIF) [file pone.0160336.s003.tif]

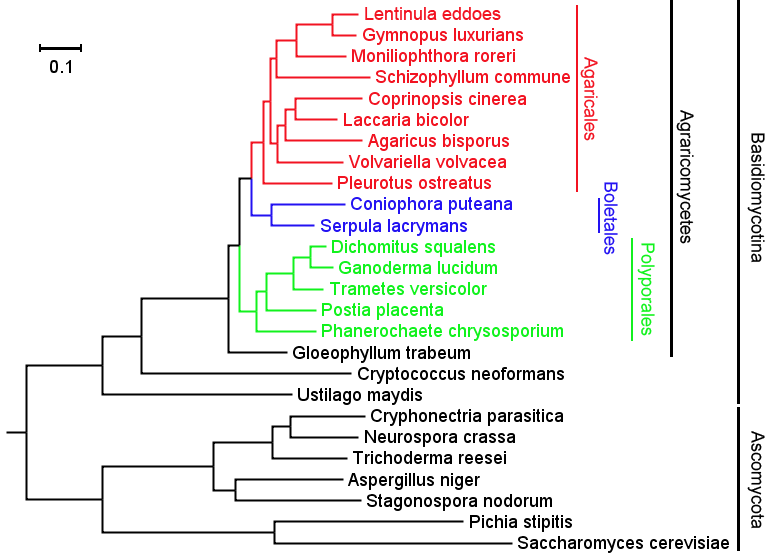

Supplement: S4 Fig — A maxlikehood phylogenetic tree of 26 fungal species was constructed using RAxML, and a bootstrap analysis with 1,000 replications was performed. All of the bootstrap values at any node were 100%. (TIF) [file pone.0160336.s004.tif]

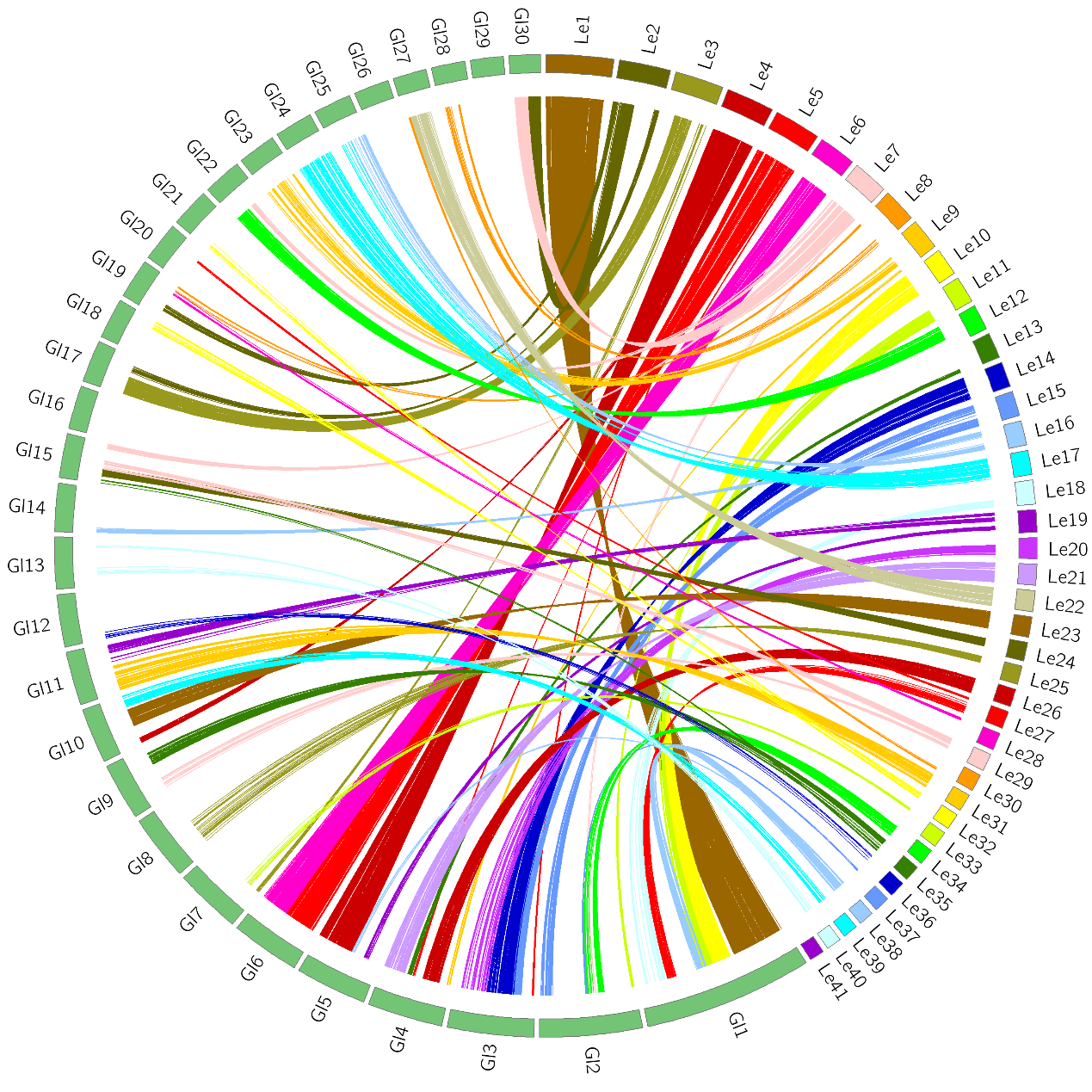

Supplement: S5 Fig — The two genome sequences shown in this picture stand for half of the genome size. (TIF) [file pone.0160336.s005.tif]
