# Supplementary material for: Genome Sequence of the Edible Cultivated Mushroom Lentinula edodes (Shiitake) Reveals Insights into Lignocellulose Degradation
Source: PLoS One. 2016 Aug 8;11(8):e0160336. doi: 10.1371/journal.pone.0160336 (PMC4976891; doi:10.1371/journal.pone.0160336)
Supplement: S1 Table — (DOCX) [file pone.0160336.s006.docx]

**Table S1. Sequencing statistics**

| **Library No.** | **Sequencing Platform** | **Insert size (bp)** | **No. Clean Reads** | **Sizes (M)** | **Coverage (X)** | **NCBI SRA Accession Number** |
| --- | --- | --- | --- | --- | --- | --- |
| 1 | Illumina Hiseq 2000 | 160±47 | 30,309,284 | 3,027 | 72.4 | SRR1917030 |
| 2 | Illumina Hiseq 2000 | 448±68 | 21,200,114 | 2,116 | 50.6 | SRR1917031 |
| 3 | Illumina Hiseq 2000 | 2370±228 | 5,848,496 | 580 | 13.9 | SRR1917032 |
| 4 | Illumina Hiseq 2000 | 4703±491 | 32,283,930 | 3,226 | 77.2 | SRR1917033 |
| 5 | Illumina Hiseq 2000 | 5255±797 | 7,450,884 | 744 | 17.8 | SRR1917034 |
