# Supplementary material for: Genome Sequence of the Edible Cultivated Mushroom Lentinula edodes (Shiitake) Reveals Insights into Lignocellulose Degradation
Source: PLoS One. 2016 Aug 8;11(8):e0160336. doi: 10.1371/journal.pone.0160336 (PMC4976891; doi:10.1371/journal.pone.0160336)
Supplement: S2 Table — (DOCX) [file pone.0160336.s007.docx]

**Table S2. Assembly statistics**

| Scaffold total number | 340 |
| --- | --- |
| Scaffold size (bp) | 41,825,817 |
| Scaffold mean length (bp) | 123,017 |
| Scaffold maximum length (bp) | 1,370,980 |
| Scaffold minimum length (bp) | 984 |
| Scaffold N50^a^ | 41 |
| Scaffold L50 (bp)^b^ | 300,654 |
| Scaffold N90^a^ | 139 |
| Scaffold L90 (bp)^b^ | 83,563 |
| Contig total number | 2,112 |
| Contig size (bp) | 40,773,698 |
| Contig maximum length (bp) | 973,258 |
| Contig minimum length (bp) | 6 |
| Contig N50a | 104 |
| Contig L50 (bp)^a^ | 106,379 |
| Contig N90^b^ | 483 |
| Contig L90 (bp)^b^ | 12,575 |

^a^ N50/N90, number of scaffolds or contigs that collectively covered at least 50%/90% of the assembly; ^b^ L50/L90, length of the shortest scaffold or contig among those that collectively covered 50%/90% of the assembly.
