# Supplementary material for: Genome Sequence of the Edible Cultivated Mushroom Lentinula edodes (Shiitake) Reveals Insights into Lignocellulose Degradation
Source: PLoS One. 2016 Aug 8;11(8):e0160336. doi: 10.1371/journal.pone.0160336 (PMC4976891; doi:10.1371/journal.pone.0160336)
Supplement: S3 Table — (DOCX) [file pone.0160336.s008.docx]

**Table S3. Gene model statistics**

| Genes Total | 14,889 |
| --- | --- |
| Alternative spliced genes | 5,333 (35.82%) |
| Complete Genes (5'M-3'*) | 14,682 (98.61%) |
| Average Gene length (bp) | 2,217 |
| Gene Size ^a^ (bp) | 32,404,000 |
| Transcripts Total | 23,633 |
| Average Transcripts length (bp) | 2,314 |
| Exons per transcript | 6.7 |
| Proteins Total | 23,633 |
| Average Protein length (aa) | 451 |

^a^ The gene size means the bp number occupied on the genome sequences by all genes.
