# Supplementary material for: Genome Sequence of the Edible Cultivated Mushroom Lentinula edodes (Shiitake) Reveals Insights into Lignocellulose Degradation
Source: PLoS One. 2016 Aug 8;11(8):e0160336. doi: 10.1371/journal.pone.0160336 (PMC4976891; doi:10.1371/journal.pone.0160336)
Supplement: S4 Table — (DOCX) [file pone.0160336.s009.docx]

**Table S4. Classification of repeated sequences**

| **Elements** | **Total number** | **Length (bp)** | **% assembled genome** |
| --- | --- | --- | --- |
| Retroelements | 6,266 | 5,143,880 | 9.73 |
| LTR | 5,787 | 4,993,363 | 9.38 |
| Gypsy | 4,146 | 3,983,513 | 7.32 |
| Copia | 1,370 | 835,975 | 1.69 |
| Ngaro | 210 | 161,358 | 0.34 |
| Pao | 61 | 12,517 | 0.03 |
| LINE | 435 | 146,448 | 0.35 |
| RTE-X | 250 | 78,420 | 0.19 |
| Tad1 | 180 | 64,854 | 0.15 |
| Penelope | 5 | 3,174 | 0.01 |
| SINE | 43 | 4,027 | 0.01 |
| Other | 1 | 42 | 0.00 |
| DNA transposons | 715 | 202,808 | 0.47 |
| Helitron | 494 | 95,065 | 0.22 |
| CMC-EnSpm | 71 | 81,544 | 0.19 |
| TcMar | 56 | 11,649 | 0.02 |
| Kolobok-T2 | 32 | 7,975 | 0.02 |
| Other | 62 | 6,575 | 0.02 |
| Unclassified | 5,981 | 2,629,457 | 6.25 |
| **Total interspersed repeats** | **12962** | **7,976,145** | **15.74** |
| Satellites | 0 | 0 | 0.00 |
| Simple repeats | 4,981 | 223,849 | 0.53 |
| Low complexity | 983 | 59,005 | 0.14 |
| rRNA | 10 | 11,175 | 0.03 |
| **Total** | **18,936** | **8,270,174** | **16.24** |
