# Supplementary material for: Genome Sequence of the Edible Cultivated Mushroom Lentinula edodes (Shiitake) Reveals Insights into Lignocellulose Degradation
Source: PLoS One. 2016 Aug 8;11(8):e0160336. doi: 10.1371/journal.pone.0160336 (PMC4976891; doi:10.1371/journal.pone.0160336)
Supplement: S5 Table — (DOCX) [file pone.0160336.s010.docx]

**Table S5. Gene model supported by hits/data from the corresponding public databases**

| Total models | 14,889 |
| --- | --- |
| Nr | 11,995 (80.6%) |
| Swissprot | 7,334 (49.3%) |
| COG | 6,756 (45.4%) |
| KOG | 7,244 (48.7%) |
| Pfam | 8373 (56.2%) |
| InterPro | 9,132 (61.3%) |
| CDD | 9.984 (67.1%) |
| GO | 7,713 (51.8%) |
| KAAS | 3,252 (21.8) |
| Total | 12,501 (84.0%) |
