# Supplementary material for: Genome Sequence of the Edible Cultivated Mushroom Lentinula edodes (Shiitake) Reveals Insights into Lignocellulose Degradation
Source: PLoS One. 2016 Aug 8;11(8):e0160336. doi: 10.1371/journal.pone.0160336 (PMC4976891; doi:10.1371/journal.pone.0160336)
Supplement: S6 Table — (DOCX) [file pone.0160336.s011.docx]

**Table S6. Resources of the other 25 fungi for OrthoMCL analysis**

| Abbreviation | Species | Downloading website |
| --- | --- | --- |
| abis | *Agaricus bisporus* | <http://genome.jgi.doe.gov/Agabi_varbisH97_2/Agabi_varbisH97_2.home.html> |
| anig | *Aspergillus niger* | <http://genome.jgi.doe.gov/Aspni_DSM_1/Aspni_DSM_1.home.html> |
| ccin | *Coprinopsis cinerea* | <http://www.broadinstitute.org/annotation/genome/coprinus_cinereus/MultiHome.html> |
| cneg | *Cryptococcus neoformans* | <http://www.broadinstitute.org/annotation/genome/cryptococcus_neoformans/MultiDownloads.html> |
| cpar | *Cryphonectria parasitica* | <http://genomeportal.jgi-psf.org/Crypa2/Crypa2.home.html> |
| cput | *Coniophora puteana* | <http://genome.jgi.doe.gov/Conpu1/Conpu1.home.html> |
| dsqu | *Dichomitus squalens* | <http://genome.jgi.doe.gov/Dicsq1/Dicsq1.home.html> |
| gluc | *Ganoderma lucidum* | <http://www.herbalgenomics.org/galu/> |
| glux | *Gymnopus luxurians* | <http://genome.jgi.doe.gov/Gymlu1/Gymlu1.home.html> |
| gtra | *Gloeophyllum trabeum* | <http://genome.jgi.doe.gov/Glotr1_1/Glotr1_1.home.html> |
| lbic | *Laccaria bicolor* | <http://genome.jgi-psf.org/Lacbi2/Lacbi2.home.html> |
| mror | *Moniliophthora roreri* | <http://www.ncbi.nlm.nih.gov/Traces/wgs/?val=AWSO01> |
| ncra | *Neurospora crassa* | <http://www.broadinstitute.org/annotation/genome/neurospora/MultiDownloads.html> |
| pchr | *Phanerochaete chrysosporium* | <http://genome.jgi.doe.gov/Phchr2/Phchr2.home.html> |
| post | *Pleurotus ostreatus* | <http://genome.jgi-psf.org/PleosPC15_2/PleosPC15_2.home.html> |
| ppla | *Postia placenta* | <http://genome.jgi.doe.gov/Pospl1/Pospl1.home.html> |
| psti | *Pichia stipitis* | <http://genome.jgi-psf.org/Picst3/Picst3.home.html> |
| scer | *Saccharomyces cerevisiae* | <http://downloads.yeastgenome.org/sequence/S288C_reference/orf_protein/> |
| scom | *Schizophyllum commune* | <http://genome.jgi-psf.org/Schco3/Schco3.home.html> |
| slac | *Serpula lacrymans* | <http://genome.jgi.doe.gov/SerlaS7_9_2/SerlaS7_9_2.home.html> |
| snod | *Stagonospora nodorum* | <http://genome.jgi.doe.gov/Stano2/Stano2.home.html> |
| tree | *Trichoderma reesei* | <http://genome.jgi-psf.org/Trire2/Trire2.home.html> |
| tver | *Trametes versicolor* | <http://genome.jgi.doe.gov/Trave1/Trave1.home.html> |
| umay | *Ustilago maydis* | <http://www.broadinstitute.org/annotation/genome/ustilago_maydis/Home.html> |
| vvol | *Volvariella volvacea* | <http://genome.jgi.doe.gov/Volvo1/Volvo1.home.html> |
