# Supplementary material for: Genome Sequence of the Edible Cultivated Mushroom Lentinula edodes (Shiitake) Reveals Insights into Lignocellulose Degradation
Source: PLoS One. 2016 Aug 8;11(8):e0160336. doi: 10.1371/journal.pone.0160336 (PMC4976891; doi:10.1371/journal.pone.0160336)
Supplement: S8 Table — (DOCX) [file pone.0160336.s013.docx]

**Table S8. Expanded Gene families of L. edodes**

| **OCG ID** | **family size of the MRCA of *L. edodes* and *G. luxurians*** | **family size of *L. edodes*** | **average protein length** | **Annotation from Swiss-Prot** |
| --- | --- | --- | --- | --- |
| OCG15 | 0 | 171 | 287 | - |
| OCG16 | 0 | 154 | 445 | Retrovirus-related Pol polyprotein |
| OCG19 | 0 | 153 | 473 | Transposon polyprotein |
| OCG53 | 8 | 109 | 542 | Transposon polyprotein |
| OCG81 | 4 | 87 | 487 | - |
| OCG44 | 5 | 57 | 770 | Retrovirus-related Pol polyprotein |
| OCG10 | 0 | 53 | 653 | Retrovirus-related Pol polyprotein |
| OCG5 | 0 | 42 | 662 | Retrovirus-related Pol polyprotein |
| OCG399 | 2 | 40 | 563 | Retrovirus-related Pol polyprotein |
| OCG1103 | 1 | 29 | 316 | - |
| OCG534 | 4 | 27 | 483 | - |
| OCG311 | 4 | 24 | 744 | - |
| OCG326 | 2 | 22 | 445 | Retrovirus-related Pol polyprotein |
| OCG1446 | 2 | 21 | 546 | Ribonuclease |
| OCG446 | 7 | 21 | 785 | Retrovirus-related Pol polyprotein |
| OCG875 | 9 | 21 | 507 | - |
| OCG4818 | 1 | 20 | 236 | - |
| OCG899 | 1 | 20 | 742 | - |
| OCG1927 | 4 | 18 | 780 | - |
| OCG371 | 3 | 18 | 181 | Chromobox protein |
| OCG3965 | 1 | 17 | 847 | - |
| OCG80 | 6 | 17 | 463 | - |
| OCG33 | 7 | 16 | 755 | Probable RNA-directed DNA polymerase from transposon X-element |
| OCG4012 | 7 | 16 | 391 | - |
| OCG5947 | 1 | 16 | 489 | E3 ubiquitin-protein ligase |
| OCG741 | 12 | 16 | 1161 | - |
| OCG137 | 10 | 14 | 1077 | Transposon polyprotein |
| OCG1466 | 4 | 14 | 777 | - |
| OCG3977 | 3 | 12 | 780 | - |
| OCG7393 | 1 | 12 | 1055 | - |
| OCG11 | 0 | 11 | 1046 | Ankyrin repeat domain-containing protein |
| OCG1663 | 1 | 11 | 406 | - |
| OCG1929 | 4 | 11 | 885 | - |
| OCG5481 | 5 | 11 | 469 | Flap endonuclease |
| OCG12 | 0 | 10 | 1545 | ATP-dependent DNA helicase |
| OCG2874 | 1 | 10 | 567 | - |
| OCG3511 | 1 | 10 | 249 | - |
| OCG682 | 2 | 10 | 1542 | ATP-dependent DNA helicase |
| OCG7132 | 4 | 10 | 251 | - |
| OCG7379 | 1 | 10 | 526 | Ribonuclease |
| OCG755 | 7 | 10 | 1124 | ATP-dependent DNA helicase |
