# Supplementary material for: Genome Sequence of the Edible Cultivated Mushroom Lentinula edodes (Shiitake) Reveals Insights into Lignocellulose Degradation
Source: PLoS One. 2016 Aug 8;11(8):e0160336. doi: 10.1371/journal.pone.0160336 (PMC4976891; doi:10.1371/journal.pone.0160336)
Supplement: S9 Table — (DOCX) [file pone.0160336.s014.docx]

**Table S9. The locus of matA and matB genes**

|  | **Gene** | **ID** | **Scaffold** | **Strand** | **start** | **end** | **protein length (aa)** |
| --- | --- | --- | --- | --- | --- | --- | --- |
| matA related genes | mip | LE01Gene00365 | LE01Scaffold0001 | + | 940470 | 942964 | 778 |
|  | HD1 | LE01Gene00382 | LE01Scaffold0001 | - | 990776 | 993254 | 808 |
|  | HD2 | LE01Gene00383 | LE01Scaffold0001 | + | 993432 | 997037 | 1132 |
| matB genes | le_pr1 | LE01Gene08679 | LE01Scaffold0053 | - | 75324 | 76997 | 467 |
|  | le_pr2 | LE01Gene08680 | LE01Scaffold0053 | - | 78206 | 80185 | 583 |
|  | le_pp1 | pp1 | LE01Scaffold0053 | - | 91613 | 91768 | 51 |
|  | le_pr3 | LE01Gene08685 | LE01Scaffold0053 | - | 92966 | 94990 | 476 |
|  | le_pp2 | pp2 | LE01Scaffold0053 | - | 92966 | 93148 | 60 |
|  | le_pp3 | pp3 | LE01Scaffold0053 | - | 100267 | 100085 | 60 |
|  | le_pr4 | LE01Gene08688 | LE01Scaffold0053 | + | 100826 | 102919 | 603 |
|  | le_pp4 | pp4 | LE01Scaffold0053 | - | 133237 | 133389 | 52 |
|  | le_pr5 | LE01Gene08702 | LE01Scaffold0053 | + | 133899 | 135923 | 585 |
|  | le_pp5 | pp5 | LE01Scaffold0053 | - | 137512 | 137685 | 57 |
|  | le_prl1 | LE01Gene02087 | LE01Scaffold0006 | - | 142727 | 144645 | 493 |
|  | le_prl2 | LE01Gene06036 | LE01Scaffold0028 | - | 75626 | 79337 | 627 |
|  | le_prl3 | LE01Gene09740 | LE01Scaffold0067 | + | 85116 | 86800 | 341 |
