# Supplementary material for: Genome Sequence of the Edible Cultivated Mushroom Lentinula edodes (Shiitake) Reveals Insights into Lignocellulose Degradation
Source: PLoS One. 2016 Aug 8;11(8):e0160336. doi: 10.1371/journal.pone.0160336 (PMC4976891; doi:10.1371/journal.pone.0160336)
Supplement: S10 Table — (DOCX) [file pone.0160336.s015.docx]

**Table S10. Gamma-glutamyl transpeptidase genes of 26 fungi species**

| **Species** | **Gene Number** | **Gene ID** |
| --- | --- | --- |
| abis | 2 | 71574, 81346 |
| anig | 3 | Aspni_DSM_1_155977, Aspni_DSM_1_157787, Aspni_DSM_1_165519 |
| ccin | 2 | CC1G_01714T0, CC1G_09313T0 |
| cneg | 3 | CNAG_01026T0, CNAG_02888T0, CNAG_03128T0 |
| cpar | 4 | Crypa2_283865, Crypa2_339703, Crypa2_342966, Crypa2_39592 |
| cput | 2 | Conpu1_89021, Conpu1_95727 |
| dsqu | 2 | Dicsq1_164807, Dicsq1_51184 |
| gluc | 2 | GL25353-R1_1, GL30295-R1_1 |
| glux | 4 | Gymlu1_152648, Gymlu1_155847, Gymlu1_160478, Gymlu1_32937 |
| gtra | 3 | Glotr1_1_110306, Glotr1_1_52828, Glotr1_1_58153 |
| lbic | 3 | Lacbi2_242632, Lacbi2_480714, Lacbi2_709265 |
| ledo | 7 | LE01Gene02157, LE01Gene02907, LE01Gene03045, LE01Gene05244, LE01Gene05245, LE01Gene06030, LE01Gene06519 |
| mror | 3 | Moror_11399, Moror_8233, Moror_903 |
| ncra | 2 | NCU01753T0, NCU04130T0 |
| pchr | 2 | Phchr2_2913202, Phchr2_2981228 |
| post | 2 | PleosPC15_2_1037212, PleosPC15_2_158524 |
| ppla | 2 | PosplRSB12_1_1129658, PosplRSB12_1_1133335 |
| psti | 2 | Picst3_58626, Picst3_78698 |
| scer | 1 | scer_s288c_YLR299W |
| scom | 4 | Schco3_2482742, Schco3_2565008, Schco3_2612027, Schco3_2635271 |
| slac | 2 | SerlaS7_9_2_1058319, SerlaS7_9_2_1079519 |
| snod | 2 | Stano2_10027, Stano2_12334 |
| tree | 4 | Trire2_5337, Trire2_5645, Trire2_69811, Trire2_80911 |
| tver | 2 | Trave1_142402, Trave1_158425 |
| umay | 3 | UM02120.1, UM02210.1, UM02291.1 |
| vvol | 2 | Volvo1_112250, Volvo1_112637 |
