# Supplementary material for: Genome Sequence of the Edible Cultivated Mushroom Lentinula edodes (Shiitake) Reveals Insights into Lignocellulose Degradation
Source: PLoS One. 2016 Aug 8;11(8):e0160336. doi: 10.1371/journal.pone.0160336 (PMC4976891; doi:10.1371/journal.pone.0160336)
Supplement: S13 Table — (DOCX) [file pone.0160336.s018.docx]

**Table S13. Lignocellulolytic genes of *Lentinula edodes***

| **Class** | **Gene name** | **EC code** | **CAZY code** | **Num** | **Genes ID** |
| --- | --- | --- | --- | --- | --- |
| Cellulase | Endo-beta-1,4-glucanase | EC:3.2.1.4 | GH5, GH7, GH9, GH12, GH44, GH45, AA9 | 21 | LE01Gene01096, LE01Gene02255, LE01Gene03338, LE01Gene04104, LE01Gene04541, LE01Gene05218, LE01Gene06603, LE01Gene08136, LE01Gene08227, LE01Gene08464, LE01Gene08665,LE01Gene08744, LE01Gene09248, LE01Gene09249, LE01Gene10266, LE01Gene12231, LE01Gene12233, LE01Gene12259, LE01Gene12659, LE01Gene13789, LE01Gene14047 |
|  | 1,4-β-cellobiosidase | EC:3.2.1.91 | GH6, GH7 | 5 | LE01Gene04089, LE01Gene04829, LE01Gene07961, LE01Gene10050, LE01Gene12864 |
|  | β-glucosidase | EC:3.2.1.21 | GH1, GH3 | 12 | LE01Gene01365, LE01Gene02578, LE01Gene02612, LE01Gene03777, LE01Gene06170, LE01Gene06300, LE01Gene07491, LE01Gene07574, LE01Gene10512, LE01Gene10634, LE01Gene11660, LE01Gene13984 |
| Hemicellulase | Endo-1,4-beta-xylanase | EC:3.2.1.8 | GH10, GH11 | 5 | LE01Gene02619, LE01Gene02639, LE01Gene03223, LE01Gene07975, LE01Gene11493 |
|  | β-xylosidase | EC:3.2.1.37 | GH3, GH39,GH43 | 2 | LE01Gene05156, LE01Gene06168 |
|  | α-glucuronidase | EC:3.2.1.131 | GH67 | 0 |  |
|  | acetylxylan esterase | EC:3.1.1.72 | CE1, CE5 | 1 | LE01Gene12496 |
|  | feruloyl esterase | EC:3.1.1.73 | CE1 | 0 |  |
|  | α-L-arabinofuranosidases | EC:3.2.1.55 | GH51, GH54, GH62 | 1 | LE01Gene08142 |
| Pectinase | pectin lyase | EC:4.2.2.10 | PL1 | 0 |  |
|  | pectate lyase | EC:4.2.2.2 | PL1, PL3, PL9 | 3 | LE01Gene04946, LE01Gene12358, LE01Gene12359 |
|  | pectinesterase | EC:3.1.1.11 | CE8 | 2 | LE01Gene06029, LE01Gene14061 |
|  | polygalacturonase | EC:3.2.1.15 | GH28 | 2 | LE01Gene06014, LE01Gene06016 |
| Lignin Oxidase | multicopper oxidase | EC:1.10.3.2 | AA1 | 14 | LE01Gene01361, LE01Gene01372, LE01Gene04008, LE01Gene04648, LE01Gene04660, LE01Gene04737, LE01Gene05032, LE01Gene06108, LE01Gene07056, LE01Gene07443, LE01Gene07758, LE01Gene08149, LE01Gene08330, LE01Gene13044 |
|  | Lignin peroxidase | EC:1.11.1.14 | AA2 | 0 |  |
|  | Manganese peroxidase | EC:1.11.1.13 | AA2 | 2 | LE01Gene08997, LE01Gene09060 |
|  | Versatile peroxidase | EC:1.11.1.16 | AA2 | 1 | LE01Gene10965 |
|  | Other peroxidase | - | AA2 | 6 | LE01Gene03530, LE01Gene03532, LE01Gene03920, LE01Gene05804, LE01Gene08720, LE01Gene08723 |
|  | Cellobiose dehydrogenase | EC:1.1.99.18 | AA3_1 | 1 | LE01Gene04232 |
| Lignin Degrading Auxiliary enzyme | aryl-alcohol oxidase | EC:1.1.3.7 | AA3_2a | 8 | LE01Gene03487, LE01Gene04010, LE01Gene04820, LE01Gene04836, LE01Gene07087, LE01Gene09147, LE01Gene09156, LE01Gene13226 |
|  | glucose oxidase | EC:1.1.3.4 | AA3_2b | 2 | LE01Gene07621, LE01Gene09425 |
|  | alcohol oxidase | EC:1.1.3.13 | AA3_3 | 4 | LE01Gene08340, LE01Gene08342, LE01Gene09192, LE01Gene10868 |
|  | Pyranose oxidase | EC:1.1.3.10 | AA3_4 | 1 | LE01Gene02678 |
|  | vanillyl-alcohol oxidase | EC:1.1.3.38 | AA4 | 2 | LE01Gene07237, LE01Gene11889 |
|  | Glyoxal oxidase | EC:1.1.3.- | AA5_1 | 3 | LE01Gene02092, LE01Gene04633, LE01Gene04793 |
|  | Galactose oxidase | EC:1.1.3.9 | AA5_2 | 2 | LE01Gene12661, LE01Gene12732 |
|  | Benzoquinone reductase | EC:1.6.5.6 | AA6 | 2 | LE01Gene05328, LE01Gene10171 |
