# Supplementary material for: Genome Sequence of the Edible Cultivated Mushroom Lentinula edodes (Shiitake) Reveals Insights into Lignocellulose Degradation
Source: PLoS One. 2016 Aug 8;11(8):e0160336. doi: 10.1371/journal.pone.0160336 (PMC4976891; doi:10.1371/journal.pone.0160336)
Supplement: S20 Table — (DOCX) [file pone.0160336.s025.docx]

**Table S20. Key words for identification of the lignocellulolytic genes by Swiss-Prot annotation**

| **Class** | **Gene name** | **CAZY code** | **Key words** |
| --- | --- | --- | --- |
| Cellulase | Endo-beta-1,4-glucanase | GH5, GH7, GH9, GH12, GH44, GH45, AA9 | Endoglucanase, Endo-beta-1,4-glucanase, Cellulose-growth-specific |
|  | 1,4-β-cellobiosidase | GH6, GH7 | Exoglucanase |
|  | β-glucosidase | GH1, GH3 | Beta-glucosidase |
| Hemicellulase | Endo-1,4-beta-xylanase | GH10, GH11 | Endo-1,4-beta-xylanase |
|  | β-xylosidase | GH3, GH39,GH43 | beta-xylosidase |
|  | α-glucuronidase | GH67 | alpha-glucuronidase |
|  | acetylxylan esterase | CE1, CE5 | acetylxylan esterase |
|  | feruloyl esterase | CE1 | feruloyl esterase |
|  | α-L-arabinofuranosidases | GH51, GH54, GH62 | alpha-L-rabinofuranosidase |
| Pectinase | pectin lyase | PL1 | pectin lyase |
|  | pectate lyase | PL1, PL3, PL9 | pectate lyase |
|  | pectinesterase | CE8 | pectinesterase |
|  | polygalacturonase | GH28 | Endopolygalacturonase |
| Lignin Oxidase | multicopper oxidase | AA1 | laccase, multicopper oxidase |
|  | Lignin peroxidase | AA2 | Ligninase, lignin peroxidase |
|  | Manganese peroxidase | AA2 | Manganese peroxidase |
|  | Versatile peroxidase | AA2 | versatile peroxidase |
|  | Other peroxidase | AA2 | peroxidase |
|  | Cellobiose dehydrogenase | AA3_1 | Cellobiose dehydrogenase |
| Lignin Degrading Auxiliary enzyme | aryl-alcohol oxidase | AA3_2a | aryl-alcohol oxidase, Pyranose dehydrogenase |
|  | glucose oxidase | AA3_2b | Glucose oxidase |
|  | alcohol oxidase | AA3_3 | Alcohol oxidase |
|  | Pyranose oxidase | AA3_4 | Pyranose 2-oxidase |
|  | vanillyl-alcohol oxidase | AA4 | Vanillyl-alcohol oxidase |
|  | Glyoxal oxidase | AA5_1 | AA5 family of CAZYme without key words “Galactose oxidase” |
|  | Galactose oxidase | AA5_2 | AA5 family of CAZYme with key words “Galactose oxidase” |
|  | Benzoquinone reductase | AA6 | AA6 family of CAZYme, None key words were used |

Note: The lignocellulolytic genes’ identification meets 2 conditions: At first, each class of lignocellulolytic gene has its corresponding CAZYme codes; Then, Key words of Swiss-Prot annotation were used for a screen, and if two or more key words were given, any one of them could be used for lignocellulolytic genes’ identification.
